# Dual Ectopic Thyroid: A Case Report

**DOI:** 10.4314/ejhs.v32i1.12S

**Published:** 2022-10

**Authors:** Fetahi Minichil, Amal Saleh Nour

**Affiliations:** 1 Department of Radiology, College of Health Sciences, Addis Ababa University

**Keywords:** Ectopic thyroid, dual ectopic thyroid, thyroglossal duct

## Abstract

**Background:**

Ectopic thyroid gland is an uncommon disorder in which thyroid tissue is located along the line of migration, dual ectopic thyroid is a rare entity where parts of the gland are located in two different locations

**Case:**

A 14-year-old girl presented with dysphagia and odynophagia of six years duration with worsening of two weeks. Physical exam showed tongue base mass. Imaging revealed two enhancing masses at the tongue base and inferior to the hyoid bone. A diagnosis of dual ectopic thyroid was made.

**Conclusion:**

Dual ectopic thyroid is a rare occurrence and proper diagnosis is essential for proper management.

## Introduction

The thyroid gland forms during the third week of embryonic development as a proliferation of endodermal epithelial cells on the median aspect of the pharyngeal floor between the first and second pharyngeal pouch (1) . At the fifth week, it migrates midline along with the anterior aspects of the hyoid bone and laryngeal cartilage. It is attached to the base of the tongue by the thyroglossal duct throughout this journey and ultimately reaches its final destination around the 7th week of gestation(1).

Ectopic thyroid is a developmental abnormality where the thyroid tissue is present anywhere along the line of migration from the base of the pharyngeal floor to the anterior neck. Estimated prevalence ranges from 1 per 100,000–300,000 with a majority of cases occurring in women (75–80%) (2,3). Nearly half of cases are asymptomatic, with the remaining presenting with obstructive symptoms of the gastrointestinal or respiratory tract during the second and third decades of life. In rare cases, patients can also present with hypo or hyperthyroid symptoms (2).

In the majority of cases, the aberrant thyroid tissue is located in one position. Dual ectopic thyroid is a rare entity, with few reported cases in the English literature. We present a case of a 14-year-old presenting with difficulty swallowing who presented to Tikur Anbessa specialized hospital. As to our knowledge, we found no case reports in Ethiopia.

## Case

A 14-year- old female presented to the pediatric outpatient department in Addis Ababa Ethiopia with a complaint of dysphagia and odynophagia of 6 years. The patient reported that she initially had difficulty of swallowing large solid foods, but her symptoms had progressed in the past 2 weeks to involve liquid foods.

On review of systems, she denies nausea, vomiting, abdominal pain, diarrhea, or weight loss. She also denies fever, shortness of breath, orthopnea, paroxysmal nocturnal dyspnea, facial or lower extremity swelling. Lastly, she denies palpitations, heat or cold intolerance, or any episodes of a panic attack.

On physical examination, vital signs were within normal range. Pertinent findings were on examination of the oral cavity, where a round well-defined red-colored mass was observed at the base of the tongue (Figure 1). No mass could be appreciated anterior to the trachea in the normal location of the thyroid gland (anterior and lateral to the second, third, and fourth tracheal rings).

Laboratory results showed that the thyroid function tests (TSH) were 6.320 uU/ml (normal range 0.27–4.2).

Neck ultrasound showed a hypo-echoic mass just below the hyoid bone. No thyroid gland lobes or isthmus could be visualized at their anatomic location. CT scan of the neck revealed two homogeneously enhancing well-defined mass lesions, the largest one at the base of the tongue and the second one just below the hyoid bone (figure 1). The bilateral thyroid lobes are not visualized in their anatomic location.

The final diagnosis was dual ectopic thyroid. The patient was on conservative management and sent home with reassurance and recommended follow-up if she developed new symptoms.

## Discussion

Ectopic thyroid tissue is from aberrance or failure of its migration usually occurring between the foramen caecum to mediastinum though there are case reports in locations like the stomach and other sub diaphragmatic organs as a result of aberrant migration or heterotopic differentiation of uncommitted endodermal cells (3).

There are very few case reports of dual and triple forms of ectopic thyroid with the present case being one of them. In dual ectopic thyroid, the commonest locations are lingual and sub hyoid (1, 3).

Ectopic thyroid is more common in females, with a female to male ratio of 4:1. It can be seen at any age particularly during childhood, adolescence, and around menopause. This is probably because of the increased demand for thyroid hormones during these stages, increasing the circulating TSH levels as well as growth of ectopic thyroid tissue. Symptoms are related to the growth of the thyroid tissue causing dysphagia, dysphonia with stomatolalia, bleeding, or dyspnea(4).

Thyroid scanning using technetium Tc-99m is the most useful tool for definitive diagnosis as it detects the presence of thyroid tissue in other sites. Ultrasound, CT scan, and MRI help for defining the extent or location. Differential diagnosis includes lymphangioma, minor salivary gland tumors, midline branchial cysts, thyroglossal duct cysts without thyroid tissue, epidermal and sebaceous cysts, hemangioma, adenoma, angioma, fibroma, and lipoma (4).

Management of the ectopic thyroid is largely dependent on the patient's symptoms and thyroid function. If the patient is asymptomatic and euthyroid, no treatment is required. For hypothyroid patients, thyroxine is first to be recommended. Surgical intervention is warranted for bulk reduction or symptoms that need surgical removal including significant dyspnea, dysphagia, dysphonia, or hemorrhage. When surgical resection is considered, patients must be informed of the likelihood of permanent hypothyroidism as 70–90% of patients do not have a functional orthotopic gland(5).

In conclusion, accurate diagnosis of ectopic thyroid tissue requires knowledge of the anatomic course and the possibility that thyroid tissue can be found along the path of migration. One must also be familiar with other neck masses that need to be excluded like thyroglossal duct cysts.

## References

[1] Santangelo G, Pellino G, De Falco N, Colella G, D'Amato S, Maglione MG (2016). Prevalence, diagnosis and management of ectopic thyroid glands. International Journal of Surgery.

[2] Noussios G, Anagnostis P, Goulis DG, Lappas D, Natsis K (2011). Ectopic thyroid tissue: anatomical, clinical, and surgical implications of a rare entity. European journal of endocrinology.

[3] Rajabi P, Eftekhari S-M, Rouhani E, Baradaran A (2018). Ectopic thyroid in stomach; a case report. Iranian journal of pathology.

[4] Adelchi C, Mara P, Melissa L, De Stefano A, Cesare M (2014). Ectopic thyroid tissue in the head and neck: a case series. BMC Research Notes.

[5] Kuramoto R, Oikawa K, Fujita K, Oridate N, Fukuda S (2013). Triple ectopic thyroid: a case report and review of literature. J Thyroid Disord Ther.

